# Intrapericardial bronchogenic cyst extirpated through median sternotomy: a case report

**DOI:** 10.1186/s44215-023-00041-6

**Published:** 2023-05-11

**Authors:** Koji Morita, Hiromu Yoshioka, Keisuke Tanaka, Junya Sugiura, Ryota Yamamoto, Yuki Goto, Koshi Yamaki, Wataru Kato

**Affiliations:** 1 Department of Cardiothoracic Surgery of Japanese Red Cross Aichi Medical Center Nagoya Daini Hospital, Nagoya, Japan; 2 Department of Thoracic Surgery of Japanese Red Cross Aichi Medical Center Nagoya Daini Hospital, Nagoya, Japan

**Keywords:** Intrapericardial bronchogenic cyst, Superior vena cava compression, Median sternotomy, Mediastinal tumor

## Abstract

**Background:**

Bronchogenic cyst sometimes occurs in the mediastinum and rarely in the intrapericardial space. When located in the intrapericardial space, the main vessels or the heart can be compressed. In addition, if it is difficult to deny malignancy or malignancy transformation potential using any modality, surgical resection should be performed.

**Case presentation:**

The patient was a 21-year-old woman with persistent symptoms similar to a cold. Enhanced computed tomography confirmed a 51 × 36 × 35-mm intrapericardial cystic structure with partial calcification. The lesion was large enough to compress and interfere with the venous return of the superior vena cava. Thrombus formation was suspected upstream of the compression site. We performed utter extirpation through median sternotomy. A histopathological examination of the surgical specimen revealed a bronchogenic cyst. The postoperative course was uneventful, and she was discharged on postoperative day 9.

**Conclusion:**

We experienced a case of total extirpation of an intrapericardial bronchogenic cyst complicated with compression of the superior vena cava. Long-term follow-up will be necessary.

## Background

Bronchogenic cyst is a foregut-derived cystic malformation of the respiratory tract. It is a rare cystic lesion with an incidence of 1 per 42,000 in the North American population [1] and mostly occurs in the mediastinum; an intrapericardial bronchogenic cyst is definitely rare. Intrapericardial bronchogenic cysts may cause compression if located close to main vessels, regardless of the size [2]. The treatment of all bronchogenic cysts is based on complete surgical excision to prevent recurrence, and the definitive diagnosis is established primarily by a histopathological examination of the surgical specimen.

We herein report a case of an intrapericardial bronchogenic cyst successfully fully extirpated through median sternotomy.

## Case presentation

**Fig. 1:**
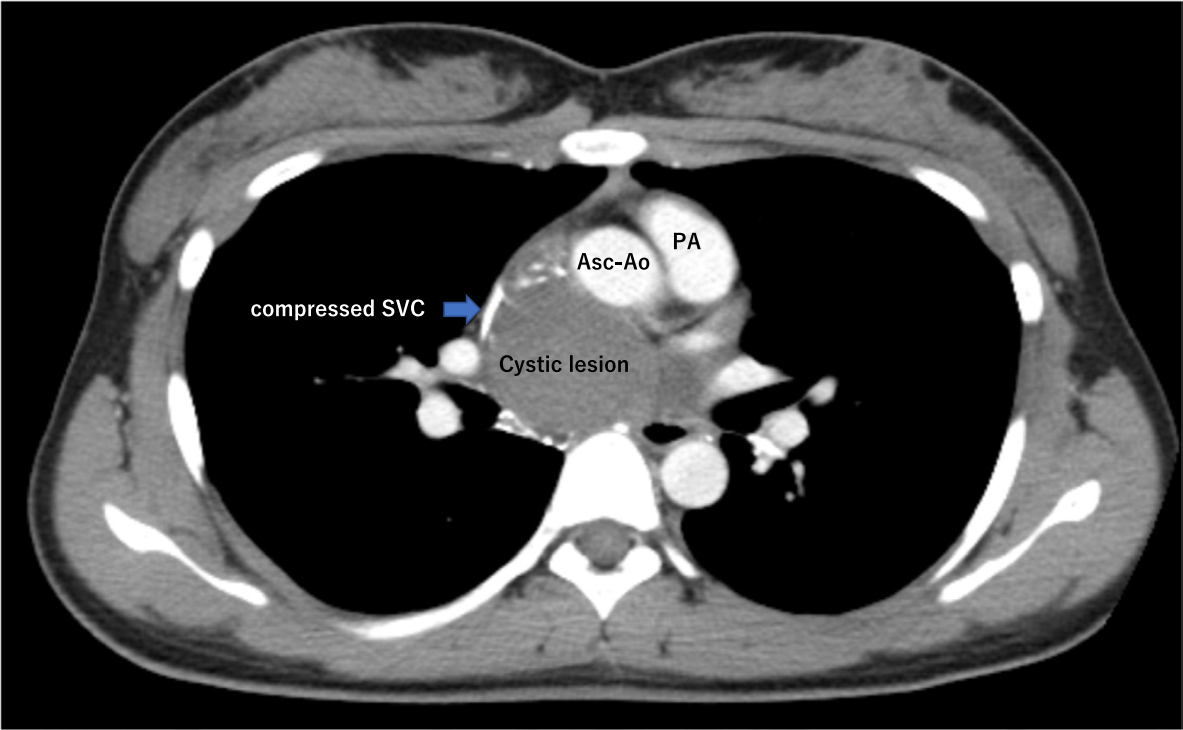
Preoperative CT scan indicating the compressed SVC

**Fig. 2:**
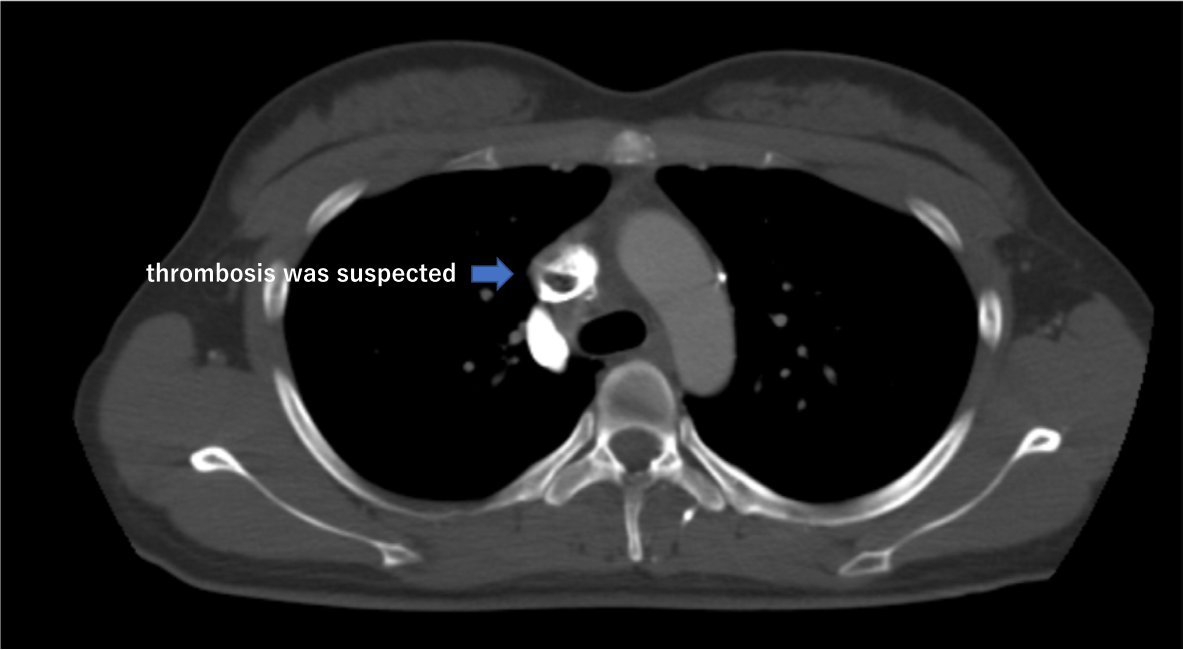
Preoperative CT scan indicating the thrombosis in the SVC

**Fig. 3:**
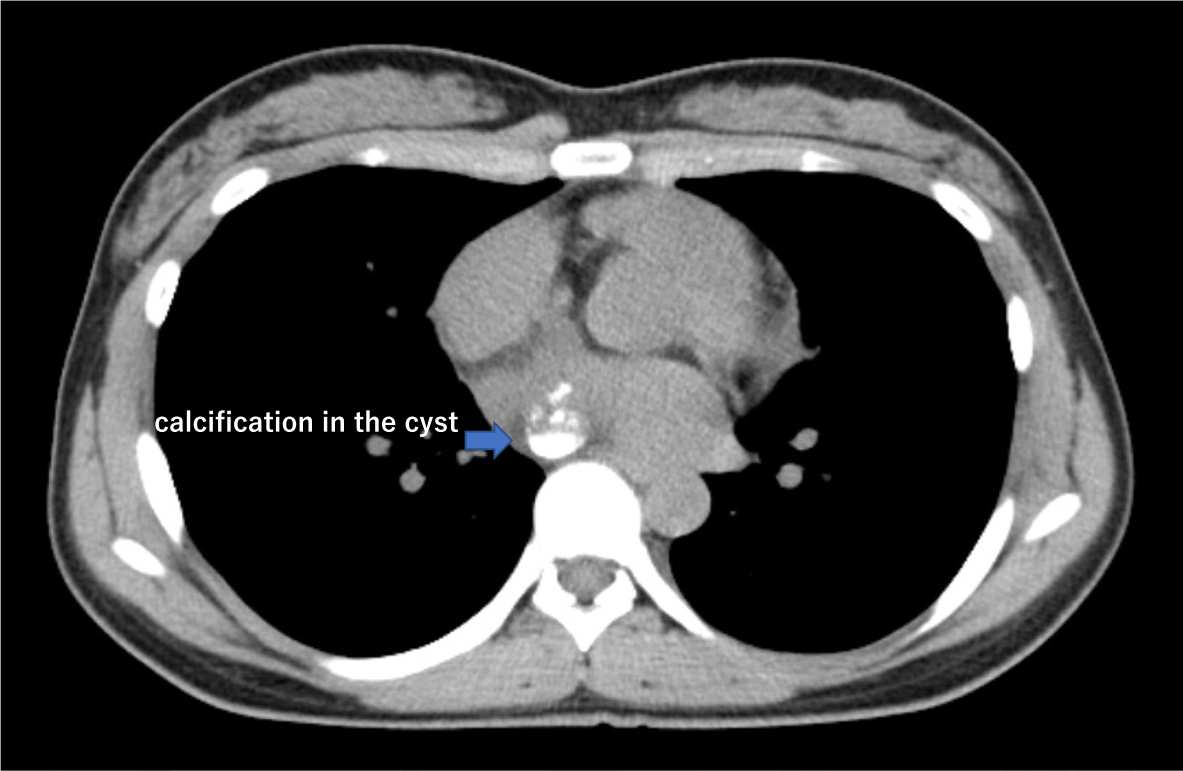
Preoperative CT scan indicating the calcification in the cyst

**Fig. 4:**
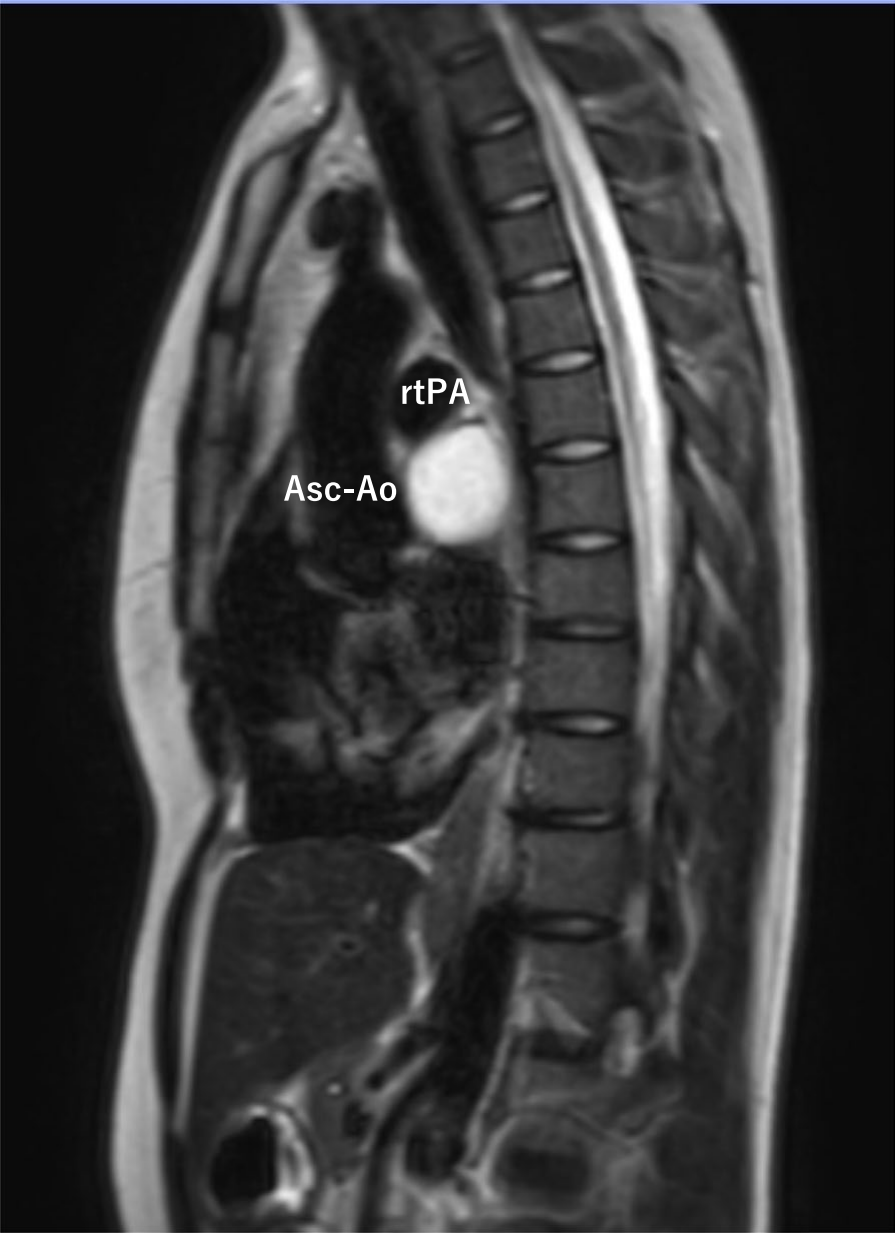
MRI indicating the location of the cyst

**Fig. 5:**
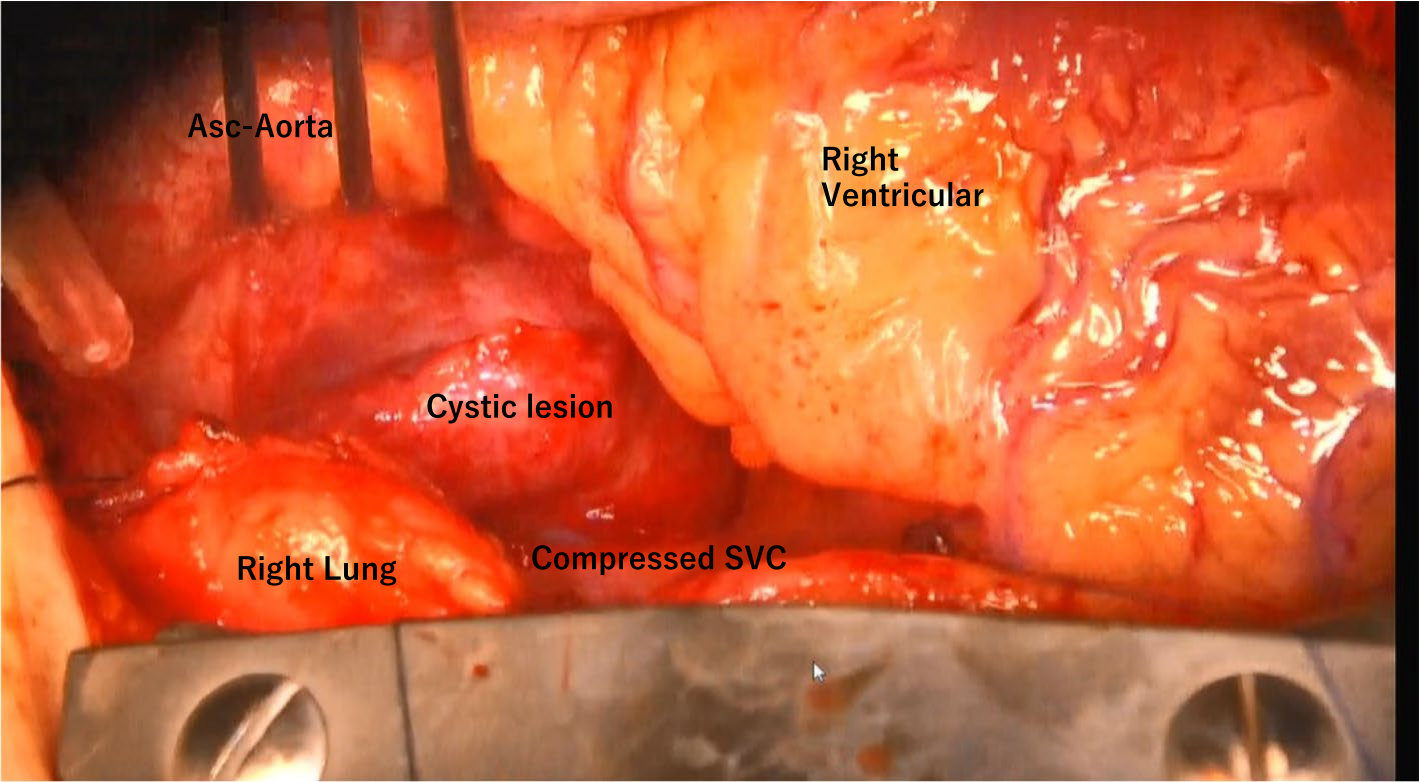
Operative findings. The cyst is located among the Asc-Ao, left atrium, SVC, right pulmonary vein, and trachea

A 21-year-old woman presented to the outpatient clinic because of a non-improving fever, cough, and general malaise. Findings on a physical examination and blood test were normal, but chest X-ray showed a widened mediastinum, and computed tomography (CT) confirmed a 51 × 36 × 35-mm cystic structure among the ascending aorta (Asc-Ao), left atrium, right pulmonary artery, and superior vena cava (SVC). The SVC was compressed by the lesion, and thrombus formation was suspected upstream of the compressed site (Figs. 1 and 2). A bronchogenic cyst was suggested by radiologists. As calcified particles were observed in the wall of the cyst, teratoma could not be excluded (Fig. 3). Magnetic resonance imaging (MRI) showed a low density on T1-weighted imaging and high density on T2-weighted imaging, so a cystic lesion was confirmed (Fig. 4). The operation was performed through standard median sternotomy. The intrapericardial lesion was firmly adhered to the posterior peripheral tissues (Fig. 5). No thrombus formation was observed in the SVC on direct vascular echography. The thrombus mimicking contrast defects observed on preoperative enhanced CT seemed to have been created by turbulence of blood flow before entering the compressed site.

**Fig. 6:**
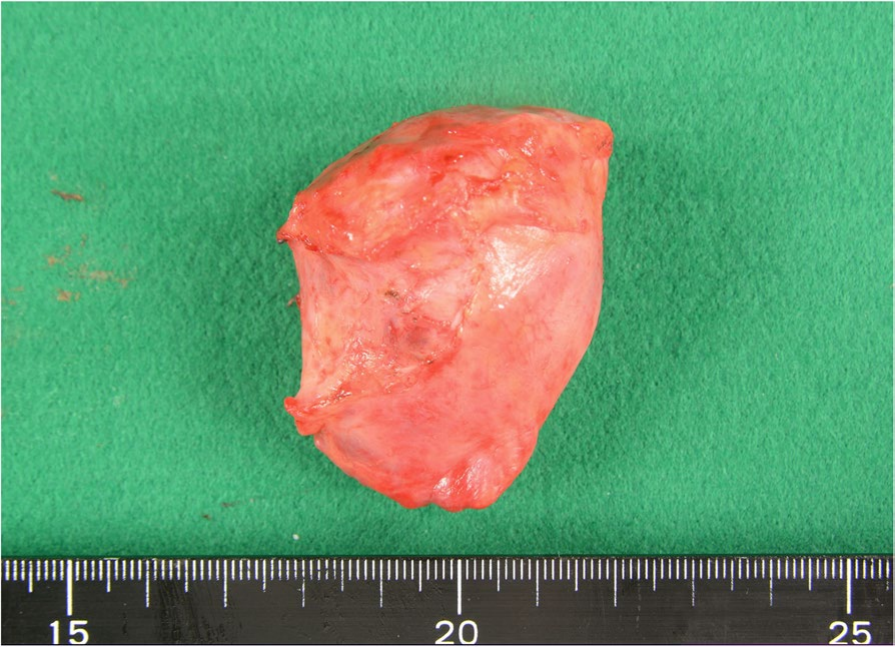
The extirpated cyst

**Fig. 7:**
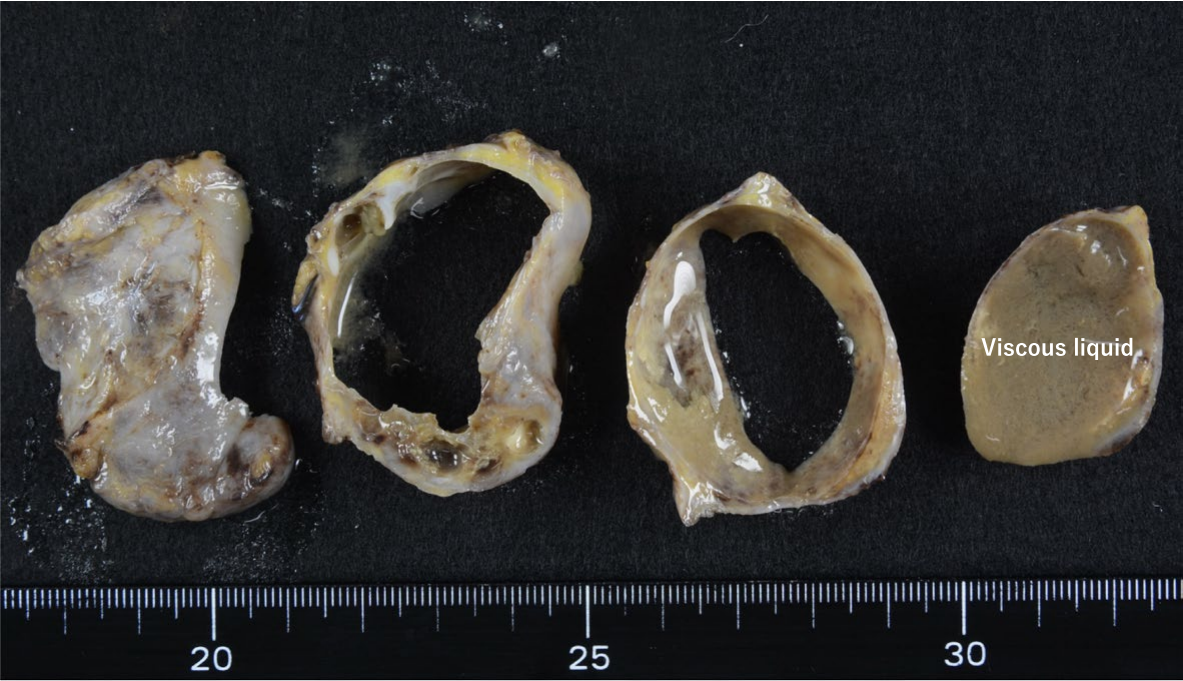
Oval and smooth in form with viscous liquid inside

**Fig. 8:**
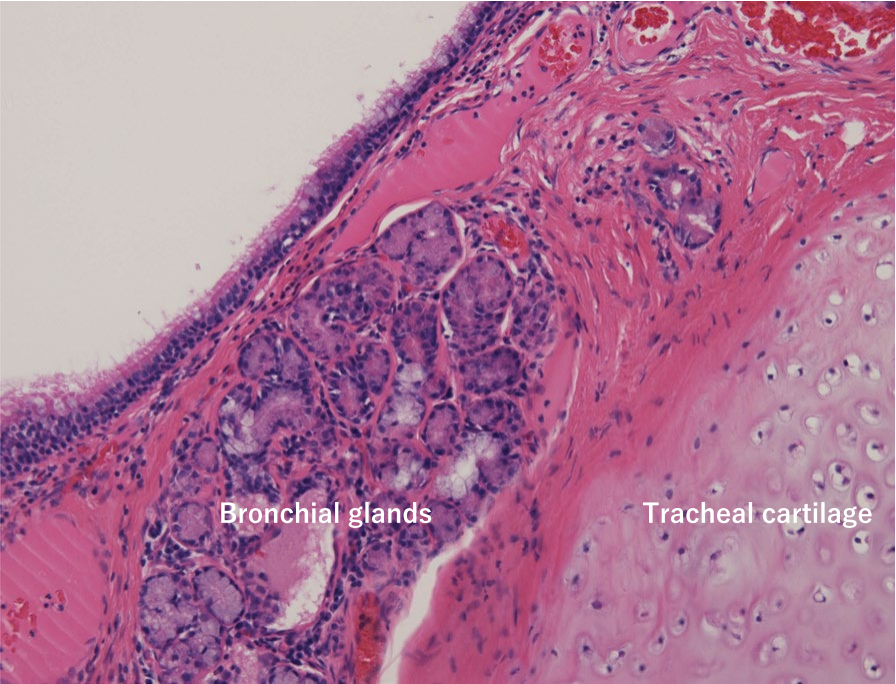
Bronchial glands and tracheal cartilage are seen, and a histopathological examination suggested a bronchogenic cyst. There was no relationship between the cystic wall and trachea

Although intraoperative bronchoscopy was employed to detect a fistula to the lesion from the trachea or bronchi, no connections were found. The severe adhesion to the posterior wall of the pericardium was carefully dissected with scissors, and complete resection was successfully performed. The SVC was completely expanded after removing the cyst, so reconstruction was not required. There was no connection with the heart, vessels, or trachea. The edge of this cyst was smooth (Fig. 6). A histopathological examination of the surgical specimen revealed it to be a bronchogenic cyst (Figs. 7 and 8).

The postoperative course was uneventful, and the patient was discharged on postoperative day 9.

## Discussion

Bronchogenic cysts are usually located within the mediastinum at an early stage of gestation or in the lung at a later stage. Their location can be anywhere along the developmental pathway of the foregut in an ectopic site, but within the mediastinum in particular, they are mainly situated at the subcarinal and right paratracheal regions [3]. According to the pertinent literature, an intrapericardial bronchogenic cyst is a rare disease, with an incidence of 1/42,000, and this is the fifth reported case from Japan. It is unusual for bronchial cysts to have a patent connection with the airway, but when present, such communication may promote infection of the cyst by allowing bacterial entry [4]. In the present case, there was no connection with the airway confirmed by intraoperative bronchoscopy.

Mediastinal bronchogenic cysts are mostly smooth, solitary, round, or ovoid masses [7]. The CT density is variable, ranging from typical water density to a high density related to blood, and they have an increased calcium content, with an MRI appearance showing an intrinsic signal intensity ranging from low to high on T1-weighted images but a high signal intensity on T2-weighted images [7]. Most frequently unilocular, they contain clear fluid or, less commonly, hemorrhagic secretions or air. These are lined by columnar ciliated epithelium, and their walls often contain cartilage and bronchial mucous glands [8].

In the present case, the tumor was firmly adhered to the posterior wall of the pericardium and not to other organs. The tumor seemed to arise from the posterior wall of the pericardium or from outside of the pericardium, extending to the intrapericardial space. There was no histological relationship between the cystic wall and the trachea, and no such marks were noted on bronchoscopy. Therefore, we suspected that this cyst was not derived from the trachea and regarded it as an intrapericardial cyst.

We were unable to distinguish the origin of the lesion, even with a histopathological examination.

In pediatric patients, bronchogenic cysts may cause life-threatening compressive symptoms [5]. In contrast, in adults, they are usually asymptomatic but rarely cause nonspecific symptoms, such as retrosternal chest pain, dyspnea, cough, a fever, and hoarseness [3]. Intrapericardial bronchogenic cysts may provoke thrombosis of the SVC, the pulmonary arteries, and veins following to compression of those. The retraction of intrapericardial bronchogenic cysts can result in SVC and pulmonary arteriovenous thrombosis [6]. These cysts sometimes cause rapid expansion, requiring emergent operation [7,8]. The treatment of patients with bronchogenic cysts depends on the symptoms at presentation and the patient’s age, and ideal approaches in asymptomatic cases remain controversial. These cysts can be distinguished from malignancy only based on resection findings and a detailed histopathologic examination. In the present case with a partially calcified cystic lesion, teratoma, which can cause malignant transformation, was not able to be excluded [9]. In this sense, early surgical intervention was justified. Severe adhesion to the surrounding tissue was expected, so we selected median sternotomy to prepare for vessel injury (Asc-aorta, pulmonary artery, or superior vena cava).

Resection must be complete because of the risk of recurrence after incomplete surgical removal [10], but it sometimes causes adhesion, particularly with inflammation or symptoms, so extirpation should be performed carefully [11]. The prognosis of bronchogenic cysts after surgical excision is reported to be excellent. However, in case of incomplete excision, late recurrences can occur [12].

In conclusion, we believe that bronchogenic cysts with compression of organs in the intrapericardial space require total resection. Surgical intervention should be considered when they are diagnosed.

## Data Availability

Raw data were generated at Department of Cardiothoracic Surgery of Japanese Red Cross Aichi Medical Center Nagoya Daini Hospital. Derived data supporting the findings of this study are available from the corresponding author K. Morita on request.
